# Effects of Different Drying Methods on the Retention of Bioactive Compounds, On-Line Antioxidant Capacity and Color of the Novel Snack from Red-Fleshed Apples

**DOI:** 10.3390/molecules25235521

**Published:** 2020-11-25

**Authors:** Aneta Wojdyło, Krzysztof Lech, Paulina Nowicka

**Affiliations:** 1 Department of Fruit, Vegetable and Plant Nutraceutical Technology, 37 Chełmońskiego Street, Wrocław University of Environmental and Life Sciences, 51-630 Wrocław, Poland; paulina.nowicka@upwr.edu.pl; 2 Institute of Agricultural Engineering, 37/41 Chełmońskiego Street, Wrocław University of Environmental and Life Sciences, 51-630 Wrocław, Poland; Krzysztof.lech@upwr.edu.pl

**Keywords:** red-fleshed apple, polyphenolics, HMF, color, kinetics, freeze-drying, microwave, vacuum, convective drying

## Abstract

The aim of this study was to determine the effect of different drying methods: convective (at 50, 60, 70 °C), vacuum-microwave (at 120, 240, 360, 480 W and 360 W with reduction to 120 W) and hybrid (convective pre-drying at 50, 60, 70 °C followed by vacuum-microwave drying at 120 W) on the quality parameters of novel red-fleshed apple fruit snacks (RFAs), such as phenolics, on-line antioxidant capacity, water activity and color. Drying kinetics, including a temperature profile of dried material, and modified Page model were determined. Freeze-drying was used as a control method. The highest content of bioactive compounds in the samples was retained following freeze-drying, then hybrid, vacuum-microwave and finally convection drying. The antioxidant capacity measured by on-line 2,2′-azino-bis(3-ethylbenzothiazoline-6-sulfonic acid) diammonium salt (ABTS), identified anthocyanins, flavan-3-ols and phenolic acid as the main compounds responsible for this activity. Unfavorable changes in color, formation of hydroxymethylfurfural (HMF) and degradation of polyphenolics were noted along with increasing drying temperature and magnetron power. The red-fleshed apple snacks are a promising high-quality dehydrated food product belonging to functional foods category.

## 1. Introduction

Apples are among the top nutritionally rated and most widely consumed fruits worldwide, and they also serve as rich sources of phenolic compounds in the human diet. The fruits play an important role both in the processing industry and direct consumption [1]. They are particularly crucial in the structure of crop production, and Poland is the third top global producer, after China and the USA.

The most important property exhibited by polyphenolics is their antioxidant activity, which in the case of *Malus* fruit is practically entirely modulated by these compounds. Regular consumption of apples prevents occurrence of many symptoms of cardiovascular diseases, oxidation of low density lipoprotein (LDL), type 2 diabetes or neoplastic diseases. It is also useful in weight management and has beneficial effects in Alzheimer’s disease patients [1]. For these reasons, food with health-promoting properties has been gaining increasing interest in the recent years. Enhanced consumer awareness means that people more often consume products rich in vitamins, minerals and antioxidants.

The huge number of apple cultivars available on the market has made producers constantly try to breed new, more attractive and more health-promoting ones. The main goal of these activities is to maintain the interest of the consumers, which also ensures sufficient demand and profitability of production. Current progress in molecular biology technologies makes it possible to understand and control the mechanisms responsible for biosynthesis pathways of nutritional and non-nutritional substances of health-promoting properties [2]. Increased demand for high quality apple fruits was conducive to the development of new breeding programs that yielded commercial red-fleshed cultivars. These cultivars occur naturally and bear fruit commonly considered to be of poor quality, as they are sour and small. For this reason, the production of consumer cultivars of improved quality has become an interesting challenge of the recent years [3]. Red color of the flesh depends on anthocyanins, which in traditional consumer cultivars are mainly found in the apple skin. The production of commercial apples with increased content of anthocyanins involves crossing wild, red-fleshed cultivars mainly of Central Asia, with white-fleshed consumer cultivars. In addition to anthocyanins, the flesh of red-fleshed apples is also rich in other polyphenolic compounds [4,5], mineral elements, sugars, acids and volatile components [3]. It is well established that consumers generally prefer red skin apples as they are associated with better taste and flavor [2,3]. Some studies [3] showed significantly higher content of total phenolic compounds, especially of anthocyanins, in red-fleshed apples (RFA) versus common apple cultivars. As mentioned above, higher content of phenolic compounds provides greater benefits to human health. 

So far, RFAs have been rarely used in the industry. Until now, literature review on RFAs shows that they are used for preparing cider [6,7], cloud apple juices [8] and beverages [9]. In addition, the effect of bagging on fruit quality during storage was evaluated [10], and freeze-dried snacks were prepared to determine the effects of food matrix on anthocyanins bioavailability [11]. Joshi et al. [12] determined the impact of drying (air-, oven- and vacuum) on the quality of cv. Redfield slices, and found that vacuum-drying offered a great potential for preserving bioactive compounds during dehydration of red apples.

So far, no efforts have been made at the production of dried apple snacks using a hybrid method of vacuum-microwave and convection drying. Therefore, novelty and the objective of the present study was to rate the usefulness of the new red-fleshed apple cultivar Trinity for the production of dry apple snacks, depending on the method and parameters of the drying process (freeze, convective (50, 60 and 70 °C), vacuum-microwave (120, 240, 360, 480 W and 360/120 W) and hybrid drying (convective pre-drying followed by vacuum-microwave (50 °C + 120 W; 60 °C + 120 W; 70 °C + 120 W)). We also determined the impact of these methods on the physical (kinetics, final moisture content, water activity and color) and chemical properties (polyphenols, hydroxymethylfurfural and antioxidant capacity (on-line, ABTS^+o^ and FRAP), sugars) of the product. In addition, principal components analysis (PCA) was performed to better understand the relationships between these parameters. Considering consumer preferences and characteristics of the raw material in the form of red-fleshed apple snack (RFAs), the selected method of drying should allow for maximum retention of the bioactive compounds and only slightly change the product appearance as compared with fresh fruit. Nowadays, or in the near future, the red-fleshed apple snacks are a promising high-quality dehydrated food product belonging to functional foods category.

## 2. Results and Discussion

### 2.1. Physical Properties of Red-Fleshed Apple Snacks

**Figure 1:**
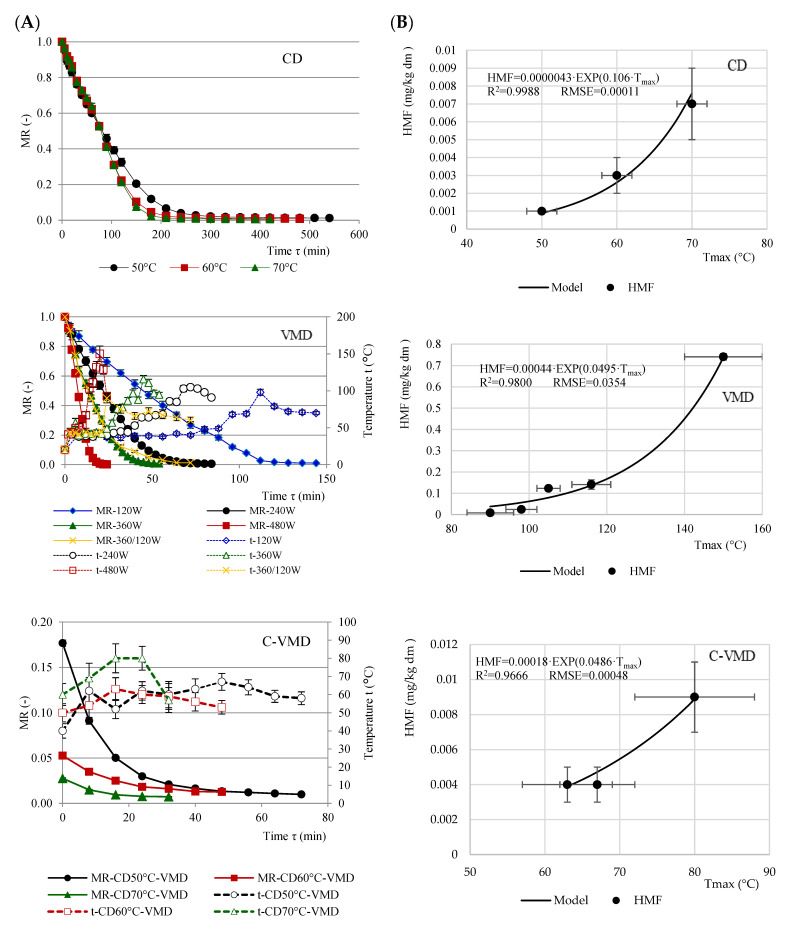
Drying kinetics: convective drying (CD), vacuum-microwave drying (VMD) and convective-vacuum-microwave drying (C-VMD) process ((**A**) as a function relationship between moisture ratio (MR) and time) and hydroxymethylfurfural (HMF) content ((**B**) as a function relationship between HMF and temperature) during drying of red-fleshed apple fruits.

Figure 1A shows drying curves indicating changes in the moisture ratio (MR) of RFAs dried using convective drying (CD) at 50, 60 and 70 °C, microwave-vacuum drying (MVD) at 120, 240, 360 and 480 W and pre drying by convective drying and finished by microwave-vacuum drying (C-MVD) at 50 °C/120 W, 60 °C/120 W, 70 °C/120 W vs. time and temperature. Drying of plant raw materials results in removal of significant amounts of water. Dry matter (DM) content in the resulting RFAs ranged from 94.09 to 98.90% and differed based on the drying conditions (Table 1). It was higher in CD 70 °C samples (97.24%) than in freeze-dried (FD) samples (97.01%), and the highest (98.90%) in VMD samples exposed to 480 W. Similar values for dried fruits were published by other authors [13,14,15].

Increasing the temperature from 50 to 70 °C during convective drying reduced the drying time by two hours (CD 50 °C = 9 h, CD 60 °C = 8 h, CD 70 °C = 7 h). Similar results were achieved during convective drying of cv. Golden Delicious apples [16]. Microwave-vacuum drying considerably shortened the drying time as compared with CD, and increasing microwave power from 120 W to 480 W made the drying six times faster (120 W = 144 min, 480 W = 24 min). Increasing microwave power was associated with increasing temperature of the sample, which for VMD at 480 W reached 150 ± 10 °C, and for 120 W 98 ± 4 °C (Table 1). Similar outcomes were described for dried jujube [17], Japanese quince fruit [14] and quince fruit [18]. Reducing the power during VMD from 360 W in the beginning to 120 W at the final stage, only slightly prolonged the drying time (6 min) vs. VMD 360 W, but allowed us to lower the samples temperature from 116 ± 5 °C for 360 W to 90 ± 6 °C for 360/120 W. Higher temperature during VMD lowers the quality of the dried material [17,19]. Hybrid drying took about half the time of convective drying. VMD after preliminary convective drying was used in many studies and yielded similar effects [20,21]. C-VMD allowed for a considerable reduction in RFAs temperature as compared with VMD. The temperature for C-VMD 70 °C/120 W reached 80 ± 8 °C, while for C-VMD 60 °C/120 W it was 63 ± 6°C. Preliminary convective drying lasted for 180 min and enabled water to be transported near the outer layers of the material by internal diffusion. Follow-up VMD heated up the entire volume of the sample, and lowered pressure-enhanced water diffusion out of the materials and its intense evaporation. In this procedure, the maximum sample temperature was much lower [20].

Table 1 presents modified Page model constants (*A*, *k* and *n*). High value of the coefficient of determination *R*^2^ (0.9715–0.9991) and low value of RMSE (0.0010–0.0343) confirmed a very good fit of the model to empirical points. The Page model was successfully used to describe drying kinetics of cv. Golden Delicious apples [16]. *A* constant denotes MR value at time 0 and it differed from 1 only for C-VMD. Shorter drying time depends on the drying rate that is associated with higher values of *k* and *n* parameters.

Water activity (a_w_) is a useful indicator of microbial growth and other chemical reactions in food products. All red-fleshed apple snacks (RFAs) had a_w_ below 0.290, which guaranteed their microbiological stability, as bacteria, yeast and molds cannot grow at such a low a_w_ [14]. In general, VMD yielded snacks with lower a_w_ than CD or C-VMD methods. If higher temperature was applied during CD, similarly to C-VMD conditions, we saw a decrease in a_w_ value. This behavior was similar to that presented in other papers, however, the lowest a_w_ was detected following FD [14,17]. Nowak et al. [22] demonstrated that water activity in guava and pineapple samples exposed to VMD should be below 0.5 to ensure shelf life of five years.

Color is a key parameter of quality for consumer acceptance, especially when final products are red. The drying method significantly (*p* < 0.001) affected lightness (L*), green-red coordinate (a*) and blue-yellow coordinate (b*) in RFAs samples (Table 1). It is essential to highlight that this is the first study on the effect of drying on RFAs color. The values of L*, a*, b* in FD RFAs amounted to 46.40, 29.34 and 7.35, respectively (Table 1). During drying, the color of the snacks changed depending on different drying methods and parameters (*p* < 0.001). In RFAs obtained by CD, VMD and C-VMD, L* value generally decreased as compared with FD, as the product samples were darker in color. The exceptions were the samples exposed to CD-70 °C and VMD-480 W. They were much brighter but at the same time less red and yellower than the other samples. In RFAs, derivatives of cyanidins, such as anthocyanin pigments, are responsible for their red color [3,7]. As compared with FD sample, the samples exposed to CD (except at 70 °C) showed a decrease in the red coordinate a* parameters. The largest change was noted for RFAs obtained by VMD: 480 W > 120 W > 360/120 W and C-VMD 50 °C/120 W. Only in RFAs obtained by CD-70 °C did we observe an increase in a* coordinators up to 31.93. 

**Figure 2:**
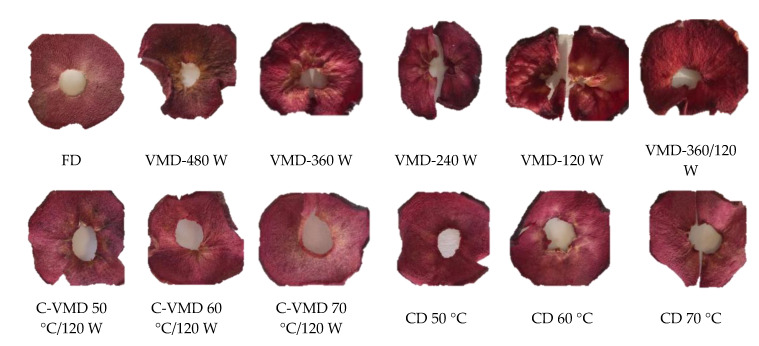
Visual dried red-fleshed apple snack.

Additionally, it was observed that samples with higher content of sugars present higher coordinated a* value, i.e., VMD 360/120 W or VMD 120 W. The highest decreased value of the blue-yellow coordinate b* obtained for RFAs sample dried by CD-50 °C/120 W, VMD-120 W and CD-70 °C, and in the other samples the b* coordinate increased up to 10.18 for VMD-480 W treatment and to 9.06 for hybrid drying (60 °C/120 W). The rest sample characterized a similar b* value for FD sample. Visual changes in color and appearance are shown in Figure 2.

The change in the color of dried plant products results from physical, chemical and biological reactions that occur during thermal processing of the material, and drying temperature may affect degradation of many bioactive compounds, including anthocyanins [13,15] responsible for red color of cv. Trinity apples. Due to high content of polyphenols, polyphenol oxidase (PPO) and peroxidase (POD), apples are particularly susceptible to an enzymatic browning. Of the analyzed drying methods, lyophilization (FD) seemed to be the most effective in retaining the color of the dried product. The effectiveness of freeze drying is due to applying low temperature that allows for high retention of bioactive compounds, comparable with raw material and minimal changes in color and chemical composition [23,24]. Apples contain large amounts of reducing sugars and vitamin C. Prolonged exposure of the raw material to hot air during CD triggers a Maillard reaction and oxidation of ascorbic acid that contribute to the formation of brown-colored reaction products. In red-fleshed apples exposed to CD, low temperature of the process resulted in stronger darkening of the samples. This might be related to longer exposure to hot air, as described by [13] during drying of cv. Annurca apples.

### 2.2. Chemical Properties of Red-Fleshed Apple Snacks

#### 2.2.1. Effects of Drying Methods and Conditions on Polyphenol Content

Analysis of RFA polyphenolics was performed by means of ultra pressure liquid chromatography with photodiode detector and fluorescence detector (UPLC-PDA-FL) and subgroups of polyphenols, such as flavonoids (as anthocyanins, flavonols, flavan-3-ols and dihydrochalcones), and phenolic acid. Table 2 presents phenolics as a sum of the singular quantified compound within a single group. The data on the presence of all identified compounds were typical for red-fleshed apple fruits and were concurrent with those reported in previous studies [3,7]. In the current study, the content of total polyphenols after FD was approx. 16 g/kg dw. Dehydration led to a reduction in total phenolics content by 7.6–45.4% during CD, by 8.6–36.6% during VMD and by 10.6–30.9% during hybrid drying (C-VMD), as compared with FD.

Data on white-fleshed apples [3,25] like Gala or Golden Delicious showed that flavan-3-ols, and especially PP, were the major group (up to 80%) of polyphenols. Analogous observations were made for the evaluated RFAs sample. In the current study, monomers, dimers and polymeric procyanidins were recognized (Table 2), which constituted almost 95% of all phenolics present in the RFAs. The content of total and individual types of flavan-3-ols in the analyzed RFAs was affected by the drying methods and their parameters. The highest content of flavan-3-ols was noted after VMD and C-VMD > CD.

Flavan-3-ol retention was significantly higher following power levels of magnetron 120, 240 or 360 W than 480 W or reduction of power from 360 to 120 W. Increasing magnetron power during VMD slightly lowered the content of polymeric procyanidins that additionally correlated with increased content of flavan-3-ol monomers. VMD at 480 W resulted in considerable frequency of breaking phenolic bounds between the units of polymeric procyanidins but at the same time in a rise in monomeric flavan-3-ols. A combination of CD and MVD at their corresponding temperatures and for a considerably reduced processing time allowed for higher retention of flavan-3-ols when compared to CD (Table 2). For CD, the strongest influence of temperature on the degradation of RFA flavan-3-ols was noticed for the sample dried at 50 °C, and the highest retention was noted for the sample exposed to 70 °C. Our observations confirmed that the time the material was exposed to the temperature was crucial for stability of flavan-3-ols. These findings corroborated those published for flowering quince [14] and for jujube [17], i.e., fruits rich in flavan-3-ol compounds.

The UPLC-PDA analysis of anthocyanins in RFA extracts revealed that cyanidins were mainly derivatives of this group of polyphenolics. Cultivar Trinity apples accumulate significantly more anthocyanins than white-fleshed consumption apples or apples with red skin [3]. The highest retention of anthocyanins in the analyzed RFAs was achieved for FD sample (350 mg/kg dw). Dehydration affected the content of anthocyanins in RFAs and their reduction by 48.4–69.0% was observed for VMD, by 27.4–43.9% for CD and by 16.5–31.9% for C-VMD. Vacuum-microwave drying with high magnetron power and high temperature stimulated destruction of anthocyanins, and reducing the power from 360 to 120 W resulted in expected preservation of these compounds. Similarly, a combination of VMD and CD ensured higher retention of anthocyanins than those methods used alone. Increasing temperature during CD resulted in linear degradation of anthocyanins and their lower content than described in the literature [26,27], even at shortened drying times. It is very important to prevent anthocyanin loss during drying, as they show the strongest antioxidant properties and other valuable biological activities [1,5].

Phenolic acid, flavonols and dihydrochalcones are the next minor groups of polyphenolics quantified in RFAs. Their concentration strongly depended on the drying method, similarly to the basic compounds (flavan-3-ols and anthocyanins). The highest content of these compounds was noted for C-VMD-70 °C, VMD 240 W and CD (50 or 70 °C). In addition, in dried RFAs, higher retention of phenolic acid, flavonols and dihydrochalcones was observed after exposure to CD-70°C. It can be concluded that thermal dehydration might be competitive to freeze-drying, especially when a combination of convective and microwave vacuum drying is applied [26].

Izli et al. [28] demonstrated variable effects of drying on the product content of polyphenols. Compared with fresh raw material, the content of polyphenols in dried product may be reduced but surprisingly it may also increase. The direction of these changes strongly depends on the type of dried material [28]. Joshi et al. [12] showed an increase in the total content of polyphenols in dried Redfield apple slices as compared with unprocessed material using vacuum drying, but the differences were not significant.

The content of polyphenols in dried red-fleshed apples depended on drying conditions. Their highest retention was achieved by application of FD, which confirmed the smallest loss of bioactive compounds, including polyphenols, when using this technique [23]. However, the main disadvantages of FD include its high costs and low efficiency associated with long processing time [15]. Convective drying is one of the most popular methods of drying plant raw materials, chiefly because the process is inexpensive. The material obtained via CD is often of worse quality than the fresh product, which is due to oxidation of bioactive compounds or other chemical changes evoked by the flow of hot air [23,29]. An alternative allowing for the production of high-quality dried products may be a combination of convective and vacuum-microwave drying (C-VMD). Proper selection of temperature and microwave power for specific materials may result in dried products with high content of bioactive substances, comparable to FD [23]. The loss of bioactive compounds may be due to increasing intracellular pressure that leads to cell rupture and release of the cell wall-associated compounds. An additional advantage of C-VMD is a relatively short dehydration time as compared with CD, which limits the duration of thermal processing leading to massive degradation of polyphenolic compounds [15,23,28].

#### 2.2.2. Effects of Drying Methods and Conditions on HMF Content

Hydroxymethylfurfural (HMF) content in processed fruit products serves as an indicator of thermal processing. HMF is an intermediate product of caramelization and Maillard reactions that is formed as a result of 1,2-enolization and dehydration of sugars. The factors that affect HMF formation include e.g., low pH, duration and temperature of the processing, water activity and the content of sugars, amino acids and other substrates in the raw material. Due to confirmed toxicity and probable carcinogenic activity of HMF, its content in food should be as low as possible [29].

Statistical analysis (*p* < 0.001) demonstrated significantly higher content of HMF in RFA samples exposed to VMD vs. the other analyzed methods. HMF concentration rose together with increasing microwave power during VMD and increasing temperature during CD. Reducing the microwave power from 360 to 120 W greatly inhibited HMF formation. A similar tendency was observed for CD, where the increase in temperature, despite shorter drying time, resulted in a greater increase in HMF. This was related to the temperature the material reached during drying, and this significantly depended on the air temperature during CD and the microwave power during VMD. Figure 1B shows the functional relationship between the temperature the material reaches and HMF content in the dried product. The nature of this relationship was exponential for all drying methods, with high coefficient of fit *R*^2^ > 0.9666 and low RMPS < 0.0354. The relationship between HMF formation and processing temperature of blackcurrant pomace was also described using an exponential function [27]. The authors identified a threshold temperature of RFAs above which rapid formation of HMF occurred. This temperature was 60 °C for CD, 100 °C for VMD and 70 °C for C-VMD.

In dried quince, HMF content ranged from 1.16 to 1.47 mg/kg dw [14], and in dried plums it reached 70.01 mg/kg dw [26], which means HMF concentration in the final RFAs was very low (max. 0.741 mg/kg dw). Michalska et al. [26] and Turkiewicz et al. [14] also demonstrated increasing HMF content along growing microwave power during drying, which closely correlated with the rise of the process temperature. Michalska et al. [26] reported incremental growth in HMF formation above 60 °C, and considerable intensification of its accumulation above 80 °C. Some previous papers [14,27] indicated that the presence of chlorogenic acid in plant materials may be conducive to HMF formation during thermal processing. In the analyzed RFAs, we found a negative correlation (*r*^2^ = −0.243) between phenolic acid (chlorogenic acid was predominant one) and HMF concentration, which suggested this acid might have only slightly affected HMF formation. The highest positive correlation (*r*^2^ = 0.988) with HMF was found for monomeric and dimeric flavan-3-ols. Low correlation coefficients were established for the content of sugars and HMF, e.g., for fructose (*r*^2^ = −0.255), glucose (*r*^2^ = 0.227), sorbitol (*r*^2^ = 0.382) and sucrose (*r*^2^ = 0.416).

#### 2.2.3. Effects of Drying Methods and Conditions on Sugar Content

Sweet taste of food largely affects consumer choices and is an important quality feature taken into account in apple breeding programs. Sugars are commonly considered the main factor conveying sweet taste of a raw material. Fructose, sucrose and glucose were the main RFA sugars, and sorbitol was the main sugar alcohol [3,30]. The content of sugars in the obtained RFAs ranged from 18.22 to 34.10 g/100 g dw and it strongly depended on the drying method: FD > CD and C-VMD > VMD (Table 1). No clear relationship was found between the drying method and the content of individual sugars. Of all fruit sugars, fructose has the sweetest taste and it considerably affects sensory properties of the product [30,31]. Aprea et al. [30] indicated that apart from influencing the taste of the product, sugars are also important for its color. In the RFAs, a strong positive correlation was found (*r*^2^ = 0.819) between fructose content and anthocyanins, suggesting a role of this sugar in pigment formation. The remaining sugars and the sugar alcohol showed weaker interactions with anthocyanins (*r*^2^ below 0.25).

### 2.3. Effects of Drying Methods and Conditions on On-Line ABTS Antioxidant Capacity, ABTS^+o^ and FRAP

Antioxidant activity cannot be unequivocally determined with only one method, as plant raw materials have complex chemical composition and the components may interact with each other [14]. For these reasons, antioxidant capacity in this study was assessed with ABTS^+o^ and FRAP assays. Contessa and Botta [25] claimed that red-fleshed apples are a rich source of bioactive substances and their amount in fresh fruit is comparable to that of blackcurrant. Our results for dried RFAs are presented in Table 2.

Antioxidant capacity of RFAs determined by ABTS^+o^ or FRAP assay increased at higher temperature or microwave power, depending on the drying method. Similar observations were made by Michalska et al. [26] for dried plums. Joshi et al. [12] also showed that enhanced drying temperature of red-fleshed Redfield apples increased the reduction strength of Fe^2+^ ions. Most likely, drying induces formation of other compounds with antioxidant capacity but not belonging to polyphenols. This phenomenon could be observed in RFAs dried using VM-480 W, where the content of polyphenolics was lower than in other samples, but it featured higher antioxidant capacity.

**Figure 3:**
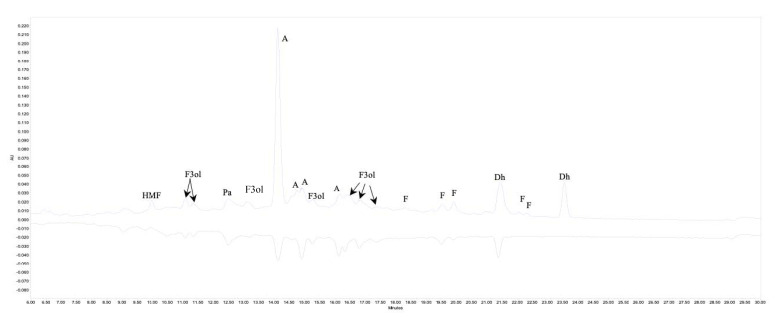
Part of chromatogram (6.0 to 30.0 min) profiles obtained before and after the derivatization analysis after ABTS^+o^ reaction for red-fleshed apples (RFAs) obtained after VMD 120 W. HMF—hydroxymetylfurfural; Pa—phenolic acid; F3ol—flavan-3-ols; A—anthocyanins; F—flavonols; Dh—dihydrochalcons.

Therefore, to better understand which compounds were responsible for the antioxidant activity, we carried out an on-line analysis with ABTS^+o^, and present an exemplary chromatogram in Figure 3.

The upper chromatogram recorded at 280 nm shows the response after passing through the first PDA detector (black as positive), and the lower one recorded at 734 nm represents the response of the eluted compounds after reaction with ABTS^+o^ after passing through the second PDA detector. The area of negative peaks on the lower chromatogram corresponds to the activity of individual compounds. The characteristic elevation of the baseline in the middle part of the upper chromatogram is caused by the presence of polymeric procyanidins, mainly RFA compounds. A mirror reflection of this elevation in the lower chromatogram after reaction with ABTS^+o^ indicates that these compounds exhibit significant antioxidant activity. Our findings are similar to those published by Tkacz et al. [32], who also observed greater activity of procyanidin polymers than monomer or other polyphenolics. The activity of phenolic acid was lower than of flavan-3-ols, which can be clearly seen in the lower chromatogram (blue as negative). The signal from anthocyanins was significant but for phenolic acid the response after the reaction with ABTS^+o^ was slight. As can be seen in the upper chromatogram, dihydrochalcone, an additional main component, showed minor antioxidant potential.

### 2.4. Principal Component Analysis (PCA)

**Figure 4:**
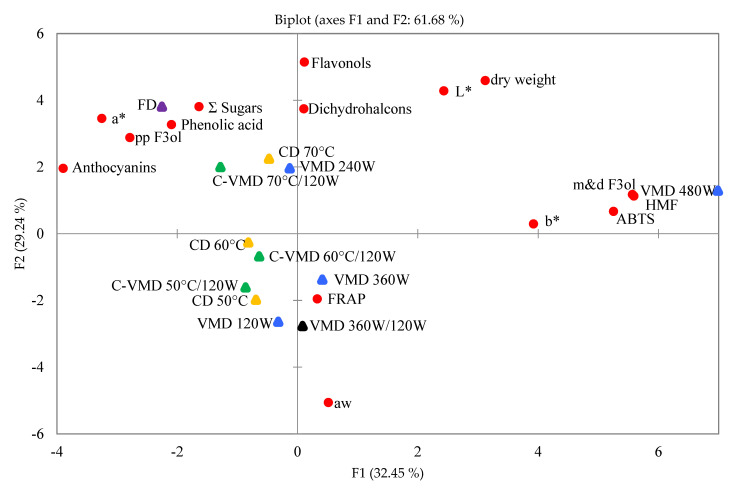
Principal component analysis (PCA) score plot showing correlations between variables and factors of RFAs.

PCA was employed to better understand the relationship between drying methods and their parameters vs. bioactive compounds and chemical composition of RFAs. Figure 4 is a bidimensional representation of all the variables and RFA samples defined by two first PCs as PC1 vs. PC2. Four PCs explained the cumulative percentage of total variation, while PC1 and PC2 explained 61.68% of total variation. PC1 (right side of the figure) explained 32.45% of the total variation, and accounted mainly for bioactive compounds (polyphenols: flavan-3-ols as monomers and dimers, flavonols and dihydrochalcones), biological activity (ABTS, FRAP), HMF and color parameters (L*, a* and b*), a_w_, dw. Antioxidant capacity (ABTS^+o^) might be conferred by monomeric and dimeric flavan-3-ols (Person correlation *r*^2^ = 0.883). Additionally, HMF closely correlated with monomers and dimers of flavan-3-ols (Person correlation *r*^2^ = 0.988), resulting in high HMF content in the snacks prepared with VMD 480 W method. PC2 explained 29.24% of total variation and accounted mainly for anthocyanins, phenolic acid and polymeric procyanidins vs. CD-70 °C, VMD-240 W and C-VMD-70 °C/120 W. In addition, the samples were characterized by high retention of anthocyanins that correlated with high values of a* parameter denoting red color hues. In addition, the snacks obtained by CD (60 °C and 50 °C) and their C-VMD and VMD-120 W counterparts showed low content of sugars, phenolics, antioxidant capacity and color indicator as compared with the other drying methods.

## 3. Materials and Methods 

### 3.1. Chemicals

All chemicals and reagents were of analytical grade and were supplied from Sigma-Aldrich (Poznań, Poland). All polyphenolic standards were supplied from Extrasynthese (Lyon, France).

### 3.2. Plant Material 

RFA fruits (10 kg) Trinity cv. were obtained from Tymbark SA company at processing maturity in October 2019 and were immediately subjected to further processing at the Wrocław University of Environmental and Life Sciences. Just before drying, red-fleshed apples were pitted and cut in slices of approximately 3 mm wide. 

### 3.3. Drying Experiments and Drying Kinetics Calculation

Red-fleshed apple fruits were dried with 4 methods: (*i*) freeze drying—FD, (*ii*) convective drying—CD at 50, 60 and 70 °C, (*iii*) vacuum-microwave drying—VMD at 120, 240, 360, 480 and 360/120 W (reduction of microwave power from the initial 360 W to 120 W to avoid overheating of the material) pressure range 4–6 kPa and (*iv*) hybrid method with a pre-treatment by CD at 50, 60 and 70 °C for 3 h and finished by vacuum-microwave drying at 120W—C-VMD. FD was used as control drying. Drying experiments were made as described previously by Wojdyło et al. [15,17] and Turkiewicz et al. [14] and were carried out in two technological replications.

Equation (1) was used for calculating drying kinetics of red-fleshed apple as a function of MR change over time.

(1) $$MR=\frac{M_{t}}{M_{0}}$$

where *M*_0_ and *M_t_* indicate moisture contents at time 0 and time t, respectively.

Preliminary tests identified modified Page model as the best one describing the drying kinetics where *A*, *n*, *k* and *t* are constants and drying time [14,17], respectively.

(2) $$MR=Ae^{-kt^{n}}$$

### 3.4. Water Activity, Moisture, Color Parameters and Sugars

Water activity meter Novasina (LabMas-terav., Lachen, Switzerland) at 20 ± 0.5 °C was used to determine the water activity of the samples. Data are the mean of three replicates.

The moisture content of the dried red-fleshed apple samples, as well as fresh fruits, was determined by drying ground samples in a vacuum dryer (SPT-200; ZEAMiL Horyzont, Krakow, Poland) for 24 h until reaching a constant weight. Data are the mean of three replicates.

The color of dried red-fleshed apple was determined using an A5 Chroma-Meter (Minolta CR300; Osaka, Japan), referring to color space CIE L*a*b*. Data are the mean of five replicates.

Sugars were determined by HPLC–ELSD (Merck-Hittachi L-7455 (Merck KGaA, Darmstadt, Germany) with ELSD 1000 (Polymer Laboratories Inc., Amherst, MA, USA)) as described previously by Wojdyło et al. [33]. Samples (approx. 2–3 g) were diluted with 100 mL of H_2_O, sonicated (Sonic 6D; Polsonic, Warsaw, Poland) during 15 min and then heated at 90 °C for 30 min with occasional shaking. Next, whole sample was centrifuged (MPW-55; Warsaw, Poland) at 19,000× *g* for 10 min, and the supernatant was filtered through Sep-Pak C-18 Cartridges (Waters Millipore, Millford, MA, USA) and through a Hydrophilic PTFE 0.20-mm membrane (Millex Samplicity Filter, Merck; Darmstadt, Germany) and used for analysis. The separation of sugars was performed on a PrevailTM Carbohydrate ES HPLC Column-W (250 × 4.6 mm × 5 mm; Imtakt; Kyoto, Japan) column. Oven temperature was set to 30 °C. The mobile phase was used with an acetonitrile water mixture (75:25, *v*/*v*) for isocratic elution; the flow rate was 1 mL/min and injection volume of 20 μL. The following parameters were used: 80 °C for an evaporative temperature, 80 °C for the nebulizer and 1.2 mL/min for the nitrogen gas flow. Quantification of sugars was performed based on calibration curves (*r*^2^ = 0.9998) of reference standards of fructose, sorbitol, glucose and sucrose injected at 1–10 mg/L (*r*^2^ = 0.999–0.997) under the same conditions, presented as sum of sugars. Data are the mean of three replicates and expressed as g of total sugar content per 100 g dw.

### 3.5. Assessment of Polyphenols and HMF

For determination of polyphenolics and HMF, the powdered samples (approx. 1 g) were taken and 5 mL of methanol/water/ascorbic acid mixture (30:68:1, *v*/*v*/*m*) with 1% hydrochloric acid was added to each sample before and after incubated overnight time (4 °C) and sonicated (Sonic 6D; Polsonic, Warsaw, Poland) for 20 min [33]. Then, the extract was centrifuged (MPW-55; Warsaw, Poland) at 19,000× *g* for 10 min at 4 °C. Finally, before analysis, the extract was filtered through a 0.20 μm hydrophilic PTFE membrane (Millex Simplicity Filter; Merck; Darmstadt, Germany) and analyzed by UPLC.

The analysis of polyphenols provided by UPLC-PDA (Aquity, Waters; Milford and Taunton, Millford, MA, USA) was provided as described previously Wojdyło et al. [34]. analysis of total polyphenols expresses as sum of dihydrochalcones (sum of phloretin and phloridzin at 280 nm), flavan-3-ols (sum of monomer, dimer, trimer at 280 nm), phenolic acid (chlorogenic acid at 320 nm), flavonols (as sum of quercetin derivatives at 360 nm) and anthocyanins (as sum of cyanidins derivatives at 520 nm). The analysis of HMF was made at 284 nm. Prior to the measurements, the equipment was calibrated using a standard quercetin-3-*O*-glucoside, (−/+)-(epi)catechin, procyanidins B1, B2, chlorogenic acid, cyanidin-3-*O*-glucoside and phloretin-2-*O*-glucoside and HMF at 1 to 5 mg/L (*r*^2^ = 0.999–0.997). Data are the mean of three replicates, and expressed as mean value as mg/kg dry weight (dw). 

For polyphenolic and HMF quantification, 5 μL of each sample was analyzed an BEH C18 column (2.1 × 100 mm, 1.7 μm; Waters Corp., Dublin, Ireland) at 30 °C with gradient elution at a flow rate of 0.42 mL/min for 15 min. The mobile phase was composed of solvent A (2.0% formic acid) and solvent B (acetonitrile) as 1% to 25% solvent B until 12 min, and then held constant to wash and re-equilibrate the column.

Analysis of polymeric procyanidins was provide by UPLC-FL using phloroglucinolysis method as described previously by Wojdyło et al. [15]. Approx. 0.05 g were precisely measured into 2 mL Eppendorf vials and freeze-dried (24 h; Alpha 1-4 LSC; Martin Christ GmbH, Osterode am Harz, Germany), then 0.8 mL of the methanolic solution of phloroglucinol (75 g/L) and ascorbic acid (15 g/L) was added. After the addition of 0.4 mL of methanolic HCl (0.3 mol/L), the vials were closed and incubated for 30 min at 50 °C with continuous vortexing using a thermo shaker (TS-100; BIOSAN., Riga, Latvia). The reaction was stopped by placing the vials in an ice bath with drawing 0.5 mL of the reaction medium and diluting with 0.5 mL of 0.2 mol/L sodium acetate buffer. Next, the vials were cooled in ice water and centrifuged immediately at 20,000× *g* for 10 min at 4 °C. The analysis of polymeric procyanidins was carried out on a UPLC-FL Acquity system (Waters Corp., Waters Corp., Dublin, Ireland) and detection was recorded at an emission wavelength of 360 nm and excitation wavelength of 278 nm. Injection of 5 μL of each sample was analyzed on an BEH C18 RP column (2.1 × 5 mm, 1.7 μm; Waters Corporation, Milford, MA, USA) at 15 °C with gradient elution at a flow rate of 0.42 mL/min for 10 min. The mobile phase was composed of solvent A (2.5% acetic acid) and solvent B (acetonitrile) as 2% B initially until 0.6 min, 9% B until 7.3 min and then held constant to wash and re-equilibrate the column until 10 min. Prior to the measurements, the equipment was calibrated using a standard (+)-catechin, (−)-epicatechin and procyanidin B1. Data are the mean of three replicates, and expressed as mean value as mg/kg dry weight (dw).

### 3.6. Determination of Antioxidant Capacity by On-Line and Spectrophotometrically Assay 

A sample for the analysis of antioxidant capacity by on-line ABTS and spectrophotometrically assay (ABTS and FRAP) was prepared as described previously [32]. Antioxidant on-line profiling by HPLC-PDA coupled with post-column derivatization with ABTS at 30 °C using CADENZA C18 column (75 mm × 4.6 mm i.d., 3 μm; Impact; Tokyo, Japan) and detection wavelength was set at 280 and 734 nm for positive and negative peaks, respectively.

Spectrophotometrically assays (ABTS and FRAP) were performed using UV-2401 PC spectrophotometer (Shimadzu; Kyoto, Japan) with protocol described previously by Tkacz et al. [32] and Wojdyło et al. [34]. For ABTS^+o^ assay, 0.03 mL of sample methanolic extract was mixed with 3 mL of ABTS^+o^ solution. The antioxidative activity was evaluated by measuring the variation in absorbance at 734 nm after 6 min for ABTS^+o^. For FRAP assay, 3 mL of reagent was prepared by mixing 20 mmol/L FeCl_3_, 10 mmol 2,4,6-Tris(2-pyridyl)-s-triazine (TPTZ)/L reagent in acetate buffer at pH 3.6 (1:1:10, *v*/*v*/*v*) with 1 mL of sample. The FRAP assay was measured at 593 nm after 10 min. Data are the mean of three replicates, and expressed as mmol Trolox (TE)/100 g sample dry weight (dw).

### 3.7. Statistical Analysis

Results are presented as mean ± standard deviation of two independent technological determinations. Statistical analysis was conducted using Statistica version 13.3 (StatSoft, Kraków, Poland) and for PCA analysis XLSTAT for Microsoft Excel2019 was used. One-way analysis of variance (ANOVA) with a significance differences below 0.001 was evaluated by Tukey test. Drying model was fitted using Table Curve 2D (Systat Software, Inc., San Jose, CA, USA). The good fitting of a model was evaluated using coefficient of determination (*R*^2^) and root mean square error (RMSE).

## 4. Conclusions

Apple snacks obtained from red-fleshed apples might be a good and healthy novel alternative to other fruits or potato snacks for young or older people. The drying methods and parameters described in the study significantly influenced the quality of RFAs in terms of their physical and chemical, especially bioactive, properties. Among applied techniques, the greatest qualitative changes occurred during CD, especially for the samples dried at 50 and 60 °C or MVD at 420 W. The changes were the smallest for FD. Hybrid drying combining CD at 70 °C followed by vacuum-microwave at 120 W allowed for better preservation of valuable bioactive compounds, especially anthocyanins, polymeric procyanidins and crucial aspects of color (a* parameter) than convective drying and vacuum-microwave drying separately. The content of HMF depended on the drying methods and their parameters, as its highest concentration was detected in RFAs after VMD-480W or CD-70 °C drying. Antioxidant activity assayed by on-line methods depended on anthocyanins, flavan-3-ols or phenolic acid but not HMF. Based on the results of this study, future investigations should evaluate in vitro anti-diabetic, anti-obesity or anti-cholinergic potential of red-fleshed apple snacks that seem to be promising high-quality, dehydrated, novel functional food.

## Figures and Tables

**Table 1:** Parameters of the model (A, *k* and n) describing the drying kinetics, final moisture content, water activity and color (L*, a*, b*) of red-fleshed apple fruit as affected by the drying method.

Drying		Parameters Model			Statistics		T_max_ (°C)	DW	a_w_	Color		
Method	Conditions	*A*	*k*	*n*	RMSE *^‡^*	*R* ^2^				L*	a*	b*
CD	50 °C	1	0.0049	1.150	0.0224	0.9961	50 ± 2e	94.26 ± 0.12a	0.286 ± 0.006b	35.24 ± 0.26a	26.31 ± 0.29f	7.74 ± 0.19a
	60 °C	1	0.0016	1.410	0.0244	0.9958	60 ± 2a	95.98 ± 0.14c	0.248 ± 0.009a	34.96 ± 0.31a	29.04 ± 0.33a	7.74 ± 0.21a
	70 °C	1	0.0012	1.480	0.0321	0.9928	70 ± 2b	97.24 ± 0.11g	0.243 ± 0.008a	52.46 ± 0.41c	31.93 ± 0.28h	5.70 ± 0.26e
VMD	120 W	1	0.0047	1.320	0.0343	0.9879	98 ± 4c	94.63 ± 0.1b	0.264 ± 0.009d	26.54 ± 0.36d	22.58 ± 0.34b	6.83 ± 0.3cd
	240 W	1	0.0108	1.370	0.0228	0.9948	105 ± 3c	96.93 ± 0.15d	0.211 ± 0.007c	35.56 ± 0.46a	27.71 ± 0.36c	8.25 ± 0.28f
	360 W	1	0.0243	1.300	0.0234	0.9945	116 ± 5h	95.89 ± 0.14c	0.249 ± 0.008a	31.71 ± 0.57b	26.85 ± 0.28g	7.72 ± 0.27a
	480 W	1	0.0181	1.830	0.0109	0.9991	150 ± 10i	98.9 ± 0.11h	0.227 ± 0.009f	52.26 ± 0.66c	21.90 ± 0.41d	10.18 ± 0.33h
	360/120 W	1	0.0370	1.150	0.0171	0.9972	90 ± 6g	94.67 ± 0.16b	0.266 ± 0.007d	28.74 ± 0.49e	22.99 ± 0.38b	7.37 ± 0.34ab
C-VMD	50 °C/120 W	0.177	0.1550	0.739	0.0044	0.9922	67 ± 5ab	95.62 ± 0.14e	0.282 ± 0.009b	33.72 ± 0.67f	24.99 ± 0.33e	6.71 ± 0.29c
	60 °C/120 W	0.053	0.1070	0.694	0.0014	0.9884	63 ± 6ab	94.09 ± 0.13a	0.281 ± 0.008b	35.35 ± 0.36a	28.84 ± 0.42a	9.06 ± 0.36g
	70 °C/120 W	0.028	0.2000	0.572	0.0010	0.9715	80 ± 8f	96.53 ± 0.15f	0.215 ± 0.009c	32.11 ± 0.44b	27.67 ± 0.44c	7.26 ± 0.39bcd
FD	-	-	-	-	-	-	26 ± 2d	97.01 ± 0.11d	0.125 ± 0.007e	46.40 ± 0.69g	29.34 ± 0.29a	7.35 ± 0.41ab

CD—convective drying; VMD—vacuum-microwave drying; C-VMD—convective-vacuum-microwave drying; FD—freeze drying; *A*, *k* and *n* are constants of the modified Page model; RMSE—root mean square errors; *R*^2^—determination coefficient; DW—dry weight [g/100 g] ± standard deviation; a_w_—water activity; L*, a*, b*—color parameters in system CIE L*a*b*; a, b, c,…—in columns, different letters mean significant differences between samples at *p* < 0.001 by Tukey test.

**Table 2:** Effects of drying method on phenolic compounds and HMF [as mg/kg dm], sugars [g/100 g dm] and antioxidant capacity [mmol Trolox/100 g dm] of red-fleshed apple fruit.

Drying		Polyphenolic Compounds							HMF	∑Sugars	Antioxidant Capacity	
Method	Conditions	F-3-ols		Dch	PA	F	A	∑PP			ABTS	FRAP
		Monomers + Dimers	Polymeric Procyanidins									
CD	50 °C	33.4 ± 1.3j	7938.3 ± 4.3l	77.1 ± 4.7l	260.7 ± 8.4a	153.4 ± 2.9l	254.6 ± 11.8d	8717.6l	<0.001 ± 0.000c	24.3 ± 2.5h	3.8 ± 0.1cd	2.7 ± 0.1d
	60 °C	23.7 ± 2.3l	12,071.6 ± 3.5h	91.5 ± 3.7f	218.7 ± 6.8g	205.3 ± 11.4i	253.0 ± 8.67e	12,863.2i	<0.003 ± 0.001c	26.7 ± 3.1d	4.0 ± 0.2cd	3.2 ± 0.2b
	70 °C	64.2 ± 2.3f	13,788.8 ± 8.6c	135.4 ± 9.5a	231.6 ± 9.2e	320.9 ± 4.9b	196.7 ± 10.5g	14,737.7b	<0.007 ± 0.002c	21.7 ± 1.8k	4.9 ± 0.1b	3.3 ± 0.0b
VMD	120 W	103.9 ± 4.1d	13,836.8 ± 6.9b	77.5 ± 6.3k	215.6 ± 6.3h	186.1 ± 10.1j	128.7 ± 14.7k	14,548.5d	0.024 ± 0.003c	18.2 ± 1.4l	3.7 ± 0.2cd	2.4 ± 0.1e
	240 W	413.1 ± 2.8b	13,108.8 ± 9.4f	116.8 ± 5.3d	253.5 ± 8.8c	353.2 ± 16.8a	158.1 ± 9.65i	14,403.4e	0.123 ± 0.010b	26.0 ± 1.5e	3.3 ± 0.1de	2.5 ± 0.3e
	360 W	273.2 ± 3.8c	13,731.1 ± 8.6d	80.3 ± 9.5h	168.7 ± 6.0l	185.8 ± 8.6k	152.3 ± 10.5j	14,591.3c	0.141 ± 0.022b	25.5 ± 1.4f	4.5 ± 0.2bc	3.0 ± 0.1c
	480 W	4758.1 ± 7.7a	8675.1 ± 8.9k	100.9 ± 9.4e	206.2 ± 7.5j	271.1 ± 4.9e	108.6 ± 9.5l	14,120.3g	0.741 ± 0.015a	24.5 ± 1.6g	7.7 ± 0.6a	3.0 ± 0.2c
	360/120 W	38.9 ± 2.5h	9420.6 ± 9.7j	77.6 ± 5.3j	181.3 ± 8.3k	225.0 ± 10.5h	180.9 ± 10.7h	10,124.2k	<0.008 ± 0.001c	22.4 ± 3.2j	3.7 ± 0.2cd	3.1 ± 0.1b
C-VMD	50 °C-120 W	92.4 ± 1.2e	10,125.2 ± 8.6i	78.5 ± 4.6i	220.9 ± 8.6f	228.8 ± 11.6g	284.6 ± 7.9c	11,030.0j	<0.004 ± 0.001c	27.1 ± 3.5c	3.5 ± 0.3d	4.8 ± 0.2a
	60 °C-120 W	34.6 ± 2.4i	12,223.0 ± 9.6g	118.0 ± 6.5c	212.8 ± 6.9i	242.3 ± 10.4f	238.7 ± 11.7f	13,069.3h	<0.004 ± 0.001c	23.89 ± 3.2i	3.7 ± 1.1cd	3.0 ± 0.2c
	70 °C-120 W	48.5 ± 4.2g	13,231.9 ± 8.5e	132.3 ± 7.3b	258.8 ± 8.3b	303.7 ± 10.7c	292.9 ± 15.6b	14,268.1f	<0.009 ± 0.002c	30.0 ± 4.1b	4.4 ± 0.5bc	3.2 ± 0.0b
FD	-	23.9 ± 2.6k	14,944.2 ± 9.7a	83.4 ± 7.5g	251.6 ± 7.9d	302.8 ± 9.6d	350.7 ± 4.9a	15,956.5a	<0.001 ± 0.000c	34.1 ± 2.4a	2.7 ± 0.3e	1.9 ± 0.01f

CD—convective drying; VMD—vacuum-microwave drying; C-VMD—convective-vacuum-microwave drying; FD—freeze drying; F-3-ols—flavan-3-ols; Dch—dihydrochalcons; PA—phenolic acids; F—flavonols; A—anthocyanins; ∑PP—sum of polyphenolic compounds; HMF—hydroxymethylfurfural; a, b, c,…—in columns, different letters mean significant differences between samples at *p* < 0.001 by Tukey test.
